# Supplementary material for: Are carbon emissions trading and green financial instruments synergistic? -Comprehensive quantitative research based on content analysis
Source: PLoS One. 2024 Mar 7;19(3):e0298601. doi: 10.1371/journal.pone.0298601 (PMC10919601; doi:10.1371/journal.pone.0298601)
Supplement: S1 Data — (ZIP) [file pone.0298601.s001.zip › Data/地级市Z_F工作报告.zip!/║┌┴·╜¡/┤≤╟∞╩╨/└┤╫╘╣1⁄2╓┌║┼í╛┬φ┐╦╩2╛▌═°í┐_2004.docx]

各位代表：

　　现在，我代表大庆市人民政府，向大会作政府工作报告，请予审议，并请各位政协委员提出意见。

　　一、2003年经济快速发展，社会全面进步，完成和超额完成主要预期目标

2003年，是大庆实施老工业基地振兴战略、全面建设小康社会的开局之年，也是经受原油减产压力、抗击非典考验和严重干旱影响的一年。我们在市委领导下，坚持邓小平理论和“三个代表”重要思想，认真贯彻党的十六大和十六届三中全会精神，自觉接受人大依法监督，积极支持政协履行职能，凝聚全市力量，解放思想，真抓实干，较好完成了政府工作各项任务。

　　———经济增长突破9％，结构调整见到成效。大庆市地区生产总值实现1125．6亿元，同比增长9．5％，超出预期目标1．5个百分点。实现地方财政总收入42．8亿元，同比增长11％。固定资产投资总额215．4亿元，同比增长6．1％。城市居民人均可支配收入达到11029．2元，同比增长12．1％。农民人均纯收入达到1980元，同比增长7．7％。城乡居民储蓄存款余额431．6亿元，比年初增加49．4亿元。城市人均住房使用面积23平方米，提高3．2％。农业结构调整步伐加快，农村经济素质整体提高。实现农业增加值34．3亿元，同比增长8％。以乳业为重点的畜牧业快速发展，实现增加值17．2亿元，占农业增加值的50．3％，提前实现主辅换位的阶段性目标；以伊利、金锣为代表的龙头企业发展到48家，农业产业化水平有所提升；绿色特色作物面积发展到276万亩，种植业结构调整力度加大；新增节灌工程600处，建设完善了绿色农产品检测和展销中心等一批功能项目，农业抗风险能力有所增强。项目建设进展顺利，工业经济发展势头强劲。开工建设120个工业项目，86个项目建成投产。石油工业保持平稳运行，实现增加值724亿元，利税690亿元，分别增长4．6％和23．3％；石化大企业实现增加值62．6亿元，同比增长14．8％；以高新区为龙头的地方工业速度与效益“双高”增长，实现增加值85亿元，利税21．3亿元，同比分别增长40．3％和28．5％，拉动全市工业增长3个百分点。第三产业质量提升，民营经济发展壮大。商业结构进一步优化，流通设施建设力度加大，各类商品批发市场达到87个，年交易额48．1亿元。实现社会消费品零售总额108亿元，同比增长12．6％，增幅居全省首位。民营经济实现增加值132．8亿元，同比增长21．5％，成为地方经济主体。旅游业实现收入7．9亿元，同比增长13．9％。

　　———深化4项重点改革，体制活力初步显现。企业改革迈出新步伐。中省直企业改革、改组、改造力度加大，企业办社会职能分离工作积极稳妥推进。地方改制企业运作趋于规范，一些遗留问题逐步得以解决。粮食企业改革成效显著，实现利润891万元，同比增长14％。日月公司成功在新加坡上市，首批募集资金1．76亿元。财政和国有资产管理体制改革取得新成效。完善政府采购和集中收付制度，开展财政投资评审，节约财政支出3．1亿元。成立国有资产经营公司，建立产权交易中心，搭建了盘活资产的平台。物业管理体制改革取得新突破。大庆物业总公司与林源物业公司完成重组和股份制改造，社会效益和经营成果显著。授予北辰、振富和热力公司特许经营权，形成了供热管理新体制。物业费、热价分别平均下调26％和15％，降低了经济发展和社会生活成本。人民医院、第二医院等事业单位财政供给、人事管理和分配制度改革取得进展。

　　———推进新一轮招商引资，对外开放步伐加快。开展10路招商，掀起对外开放新热潮。全市引进项目297个，到位资金45亿元。创新人才政策，改善人才环境，引进各类人才1980人。毛毯、亚麻纱和肉制品等产品打入俄罗斯、中东等国际市场，全市对俄贸易额完成3120万美元，外经贸进出口总额达到7444．2万美元，同比增长29．6％。

　　———“四百”工程加快推进，城市面貌变化显著。实施“百项建设”，投资79亿元，开工建设179个城建项目，施工面积255万平方米。实验中学迁建、西城区污水处理厂等15项市级重点工程开工建设，八一农大新校区、祥阁学校等18项市级重点工程交付使用。新建县、乡、村公路669公里，城市公共交通稳步发展。推进“百区改造”，完成45个老居住区1296栋住宅楼改造，建成7处共40公顷的休闲广场，老区旧貌换新颜。开展“百湖治理”，综合治理碧绿湖、三永湖，万宝湖和黎明河水体污染问题基本解决。加快“百园建设”，启动让胡路区生态园、城市森林公园等20个生态园建设，主城区退耕还林2万亩，新建城市绿地1400公顷，城市绿化覆盖率达到33．1％，完成造林和生态林草建设52．1万亩。

　　———地企“五联”更加深入，发展合力明显增强。按照小油田联开、高新区联办、城市环境联建、招商引资联手、社会稳定联抓的原则，创新发展地企关系，实现优势互补、合作双赢。开发小油田实现利税3．5亿元。龙化新实业、志飞化工等一批存续改制企业入驻宏伟、兴化园区。联合实施老区改造、生态建设和城市基础设施建设，城市功能得到增强，环境质量明显提高。整合人才劳动力市场，形成了全市统一、竞争有序的人才市场。整治油田治安秩序，组织油田重点区块专项治理，查处侵害油田违法犯罪案件2049起。完成石油管理局900万平方米历史遗留房屋确权工作，办理大企业职工房屋产权认证2．2万件，激活了房地产市场。

　　———“大文化”建设扎实有效，精神文明成果丰硕。科技创新能力不断提高。获得省政府科学技术奖27项，组织实施科技计划项目466项，高新技术企业达到249家。社会事业全面进步。引进、整合、提升7所高校，大庆中学等4所学校通过省级示范高中复检，高考成绩创历史最好水平。万众一心，众志成城，抗击非典斗争取得阶段性重大胜利。公共卫生体系和疾病预防控制体系建设得到加强，母婴安康工程和农村中医“十百千万”工程收到较好效果。话剧《特殊故事》、舞剧《关东女人》等一批文艺精品在国家和省获奖，举办第三届大庆之冬艺术节，开展文化“双百”进社区等系列活动，群众文化生活丰富多彩。成功举办八运会等大型赛事，群众性体育运动广泛开展，竞技体育取得较好成绩。开展“百事剖析、百题突破”主题教育，组织纪念铁人诞辰80周年系列活动，推进“文明社区”、“文明单位”、“文明窗口”创建，强化“四五”普法教育，市民素质和城市形象整体提升。

　　———着力筑牢“三大基石”，社会形势保持稳定。千方百计扩大就业。健全市、区（县）、街道（乡镇）、社区四级就业组织网络，开展就业培训1．5万人次，新增就业岗位4．6万个，安置下岗失业人员4．3万人，城镇登记失业率下降到4．2％。社会保障体系不断完善。城市居民低保扩面新增1．5万人，发放低保资金3458万元。社会保险覆盖面持续扩大，各类保险金发放率100％。社区建设进一步加强。消防、邮政、体育向社区延伸，服务网点发展到4563个，安置下岗失业人员1．5万人，社区功能逐步完善。积极推进防范管控社会化，严厉打击各种违法犯罪，强化重点行业和重点区域的安全管理，依法妥善解决群众信访和热点难点问题，打击“法轮功”、“实际神”等邪教组织，确保了政治安定和社会稳定。

　　———实行“三先、四零”政策，发展环境全面改善。完善配套“先投后转、先租后卖、先借后还”和“零审批、零收费、零等待、零租金”的投资政策，降低了企业投资成本，经济发展环境实现创新。落实公示制和承诺制，实施阳光收费，政务公开力度加大。建立电子政务信息网，政务信息化步伐加快。规范政府办事程序，下放审批权限，减少审批环节，依法行政稳步推进。实施专项资金和经济责任审计，立案查处公务员违纪违法案件417件，“两风”建设得到加强。

　　此外，新闻出版、民族宗教、文史档案、外事侨务、驻外接待、技术监督、计划生育、统计、人防、国防后备力量建设等项工作，也都取得了新成绩。

　　各位代表！过去一年我们遇到的困难比预料要大，取得的成绩比预期要好。这些工作成果，体现了市委的正确思路和坚强领导，饱含着几大班子的群策群力和共同奋斗，凝结着大企业的密切协作和奋力拼搏，展示了全市上下奋发有为的创新意识和创业能力，奠定了加快大庆发展的坚实基础。在此，我代表市政府，向所有为大庆发展做出贡献的人们表示衷心感谢！

　　各位代表！过去一年取得的成绩令人鼓舞，但前进道路上还有不少矛盾和问题。主要是：接续产业发展需要进一步加快，经济结构调整任务艰巨；优势资源开发需要进一步延伸，油气、石化和农产品加工等产业链条深度不够；农业产业化水平需要进一步提高，农民收入增长不快；城市功能需要进一步增强，城市规划、建设、经营和管理的良性互动还未完全形成；就业岗位需要进一步开发，社会稳定形势依然比较严峻；官僚主义、形式主义和消极腐败现象需要进一步消除，一些部门和个别工作人员服务意识不强，执法水平不高，抓落实力度不够。这些问题，我们要高度重视，深入分析，坚持用改革和发展的办法，在今后的工作中认真加以解决。

　　二、把握历史机遇，明确发展任务，抓好牵动全局的大事实事

　　2004年，全市上下，面对加快完善市场经济体制和振兴老工业基地的战略任务，既要抢抓发展新机遇，也要积极应对新挑战。中央振兴老工业基地的政策，会不会充分利用，最大限度得到倾斜；国家确定老工业基地调整改造项目，能不能抢得上，争得来；深化企业改革，敢不敢在产权制度上大胆突破，实现创新发展？需要我们进一步开拓进取，乘势而上。

　　2004年，全市上下，面对保持较高经济增长速度、力争达到全省平均发展水平的艰巨任务，既要勇担压力，更要深挖潜力。今年原油产量计划下调210万吨，预计将减少增加值32亿元，影响市本级财政收入4000万元。但同时，油田公司靠投资拉动、外围开发、拓展市场，预计实现增加值702亿元，同比增长1．7％；中省直石化企业新上项目、挖潜增效，预计实现增加值75亿元，同比增长30％；存续企业深化改革、释放潜力，预计实现增加值74亿元，同比增长13．6％；地方经济增量提质、快速发展，预计实现增加值313亿元，同比增长22％。我们必须把握市情，坚定信心，努力实现经济快发展、大发展。

　　2004年，全市上下，面对加快建设高科技现代化城市、全面建设小康社会的历史任务，既要解放思想，创新发展思路和目标，更要突出重点，扎扎实实抓好事关全市发展大局和为民利民的大事实事。今年政府工作要按照市委总体部署，坚定不移地抓好推进落实。在力争原油稳产和长期开发的基础上，大力发展非油产业和非国有经济。主要预期目标是：大庆市地区生产总值增长8％以上，力争达到全省平均水平。一、二、三产业增加值分别增长16％、7％和16％。全社会固定资产投资增长5％以上，全市地方财政总收入按可比口径比上年预算增长11％以上，城市居民人均可支配收入增长8％以上，农民人均纯收入增长3％以上，城镇登记失业率控制在5％以内。

　　完成今年发展目标，要围绕以下6个方面突出抓好20项大事实事。

　　工业经济要重点建设石油、石化、新材料、农产品深加工和医药五大基地，支持服务大企业，开发建设大项目，培育发展优势产业，力争实现增加值950亿元，同比增长7．1％。

　　1、抓好促进石油石化大企业发展的油化立市大项目。石油、石化大企业是大庆经济的主体，服务大企业可持续发展始终是我们的首要任务。支持油田公司加大勘探力度，加快技术创新，加强成本管理，实现降产不降增加值，创建“百年油田”，为大庆经济转型提供更长的机遇期。支持石化大企业开发一批支柱型、主导型、高科技型大项目，带动全市经济增量提速。重点帮助大企业完善建设120万吨加氢裂化、100万吨重油催化、80万吨乙烯改扩建、30万吨聚丙烯、10万吨ABS专用树脂改造等一批大项目。支持大企业加快论证开发30万吨复合肥扩建至100万吨、80万吨聚乙烯、80万吨尿素等一批潜力可观的大项目，并积极帮助筛选项目，协调上报立项，争取更多项目列入国家老工业基地振兴计划。

　　2、抓好做强地方工业的“百项工程”。今年是“项目开发年”，全市地方工业重点推进121个项目，计划总投资107亿元。新建项目103项，包括20万吨醋酸、15万吨不锈钢、100万张皮革、20万立方米人造高密度板等项目；续建项目18项，包括鲍斯50万吨生物菌肥、日月3．8万吨大豆蛋白、晟凯300万条毛毯等项目。加大对具有成长潜力企业的扶持力度，培育出一批地方骨干企业，争取再有1－2户企业上市融资。研究开发一批高新技术产业项目，包括5000吨碳纤维、10万片砷化镓、3000万支脑心康冻干粉针等项目，提高地方工业的科技水平。地方工业增加值实现110亿元，增长40％以上。

　　3、抓好以高新区为龙头的园区建设。高新区加压力、提速度、增项目、扩总量，重点抓好总投资34亿元的18个大项目开工建设，推进总投资13亿元的6个续建项目，确保工业增加值增长40％以上。抓好创业广场建设，完善园区功能。提升杜尔伯特德力戈尔工业园、龙凤万宝工业园，加快基础设施配套，赋予市级管理权限，实行封闭运行。启动精细化工园二期工程，力争再引进30户企业。新建肇源皮革城、肇源港桥工业园、林甸食品工业园、红岗生态工业园、林源医药中间体专业园5个专业园区。

　　农业经济要大力推进农转牧，加快结构调整，建立和巩固牛经济为主体、猪鹅经济为两翼、畜牧业为主导产业的农村经济新格局。全市畜牧业增加值实现21．6亿元，占农业增加值的53％，拉动农业增长10个百分点。

　　4、抓好推进农转牧的牛猪鹅经济增量提质。突出发展奶牛饲养，奶牛存栏达到27万头，鲜奶产量达到80万吨以上。生猪、肉牛、肉羊、大鹅饲养量，分别达到250万头、46万头、188万只和900万只，比上年大幅增长。健全完善畜禽疫病防疫体系，重点疫病免疫密度确保达到100％。推广先进技术，奶牛单产达到5吨以上。成立乳业风险金管理委员会，完善乳业市场风险防范机制。扶持引进乳业龙头企业，支持伊利、惠尔康等企业上项目、快发展，努力形成群龙竞舞的生动局面。抓紧研究论证40万头绿色无抗奶牛基地、550万头生猪、3000万只大鹅等一批大项目，争取早开发早启动。

　　5、抓好促进农业增效农民增收的基本旱涝保收田建设。抓住国家实施优质粮食产业工程的有利契机，用3－5年建成400万亩左右旱涝保收、稳产高产基本农田。重点搞好中小型水利设施建设，扩大有效灌溉面积，提高排涝抗旱能力。大力发展特色绿色种植业，形成粮食、亚麻、蔬菜、大豆、马铃薯5个优势产业带；发展优质饲草饲料产业，全市青贮饲料种植面积达到80万亩，逐步把大庆建成全国优质饲料饲草基地。粮经饲比例调整到50∶35∶15，保护地粮食单产平均达到500公斤以上，经济作物亩收入平均达到500元以上，农民收入明显增加。400万亩以外的耕地，宜林则林、宜草则草，开发多种经济产业，建设一批大农场、大草场和大林场。

　　第三产业要以高带低、以新型带传统、以人流带物流，整合商业资源，优化商业结构，发展现代流通业，使之成为拉动经济增长的重要力量，力争实现增加值155亿元。

　　6、抓好培育商贸中心区的商业街改造。以改造建设经六街为重点，带动石油学院商业街、让胡路区商业街和东湖商业街等15条商业街提档升级。经六街要建成一流水平的全封闭、高档次的步行商业街，发展优质特色服务，营造浓厚商业氛围，并以此带动经四街、经五街和东安市场的改造，提升中心商业区水平。发展超市、仓储、连锁等新型业态，改造建设汽配城、建材城等现代批发大市场，在交通枢纽和主要物资集散地建立第三方物流园区，增强大庆商业的凝聚力和辐射力。

　　7、抓好以绿色温泉城为重点的旅游品牌建设。围绕建设区域旅游中心城市目标，深入研究开发旅游资源，建设特色精品项目，推动大庆旅游业快发展、上档次。在完善石油文化、湿地风光、草原风情游的基础上，重点启动建设绿色温泉城。今年要高起点做好项目规划设计，采取招商引资、大企业参与、社会投入等多种办法进行启动，最终开发建设成集大型生态园、温泉浴场、星级宾馆、高档别墅以及金融、购物、信息、餐饮、娱乐于一体的高标准综合旅游城。进一步整合大庆旅游资源和市场，促进旅游项目的深度开发。

　　8、抓好覆盖大庆的区域互联网建设。积极稳妥推进网络资源优化配置，整合网络资源，实现互联互通。重点以有线电视宽带网为基础，以企业化方式进行整合，建立统一的覆盖全市城乡的宽带网，并在此基础上建立公用信息平台，开展政府、企业和社会的信息服务。壮大网络经济，发展信息产业，形成新的三产业态。

　　城市和生态建设要按照人与自然和谐统一的要求，突出完善城市功能，推进生态园林城市建设，高起点规划城市，高标准建设城市，高质量整理城市，高效能经营城市，全面加快城市化步伐。

　　9、抓好实施城市东移北扩的五湖新区开发建设。重点启动“五湖新区”开发建设工程。一次性规划，分批次整理，分阶段开发。今年要完成45平方公里区域控详性规划编制，延伸经九街使之与301国道贯通，推进基础设施配套建设。与此同时，加快引进大学和科研院所的步伐，开工建设一批与之相配套的高级住宅区、商贸服务区、文化功能区项目，完成建筑面积50万平方米以上。争取用3－5年时间，把五湖新区建设成为具有北国水乡特色的生态新区、功能新区、经济新区。

　　10、抓好构建城乡交通骨架的重点路桥工程。市区抓好301国道绕行线、萨大路跨线桥、西干线、西苑街、经九街等骨干道路建设改造工程，构建“三横四纵一绕”城市交通骨架。继续实施通县、乡、村公路工程，新开工建设公路1014公里，实现41个乡镇以高等级路面相通、368个行政村完成路面硬化。争取到2007年，再新建通县、乡、村公路1817公里，市与县、县与县之间全部以二级以上公路相连，乡乡通高等级路面，村村通硬化路面，在全省率先达到村村通。同时，论证哈大公路改造提升、大庆机场、铁路枢纽站、公路客运枢纽站、环城轻轨等项目。

　　11、抓好改善生态环境的闭流区、三化区综合治理。启动主城区5000平方公里闭流区生态工程，分区做出规划、做成项目，用8－10年时间使闭流区生态基本恢复。启动果午泡、乘风湖、东风泡、董家泡、龙凤湿地自然保护区综合治理工程，逐步使主城区主要湖泊连通。搞好三化区治理，完成油田产能区植被恢复30万亩。实施主城区立体绿化建设。以点带面，新建12个生态园，在高速公路口和奔腾转盘以西三角带，建设进入东西主城区的绿化精品景观。以带补面，新建完善世纪大道、西干路等30条绿色生态长廊。以片扩面，再新建绿地1400公顷，退耕还林2万亩，建成区绿化覆盖率达到34．1％，人均公共绿地7．5平方米。继续抓好堵西北风口、城防林、农防林等重点林业项目，完成植树造林22万亩。

　　12、抓好提升城市形象的老城区整理。坚持“以人为本、治城为民”新理念，为市民建设舒适家园。继续推进百区改造工程。今年改造35个老居住区，对364栋住宅楼实施平改坡，力争用5年时间，完成全市所有老居住区改造任务。推进外来人口居住区建设和城中村、棚户区整体改造。在让胡路区盖家屯、中桥路公园桥规划建设外来人口居住区，解决流动人口集中居住管理问题。以红旗村、青龙山地区、铁人桥平房区为试点，认真做好规划设计、居民动迁、基础设施建设等项工作，推进“消灭城中村”工程，把整个城市变美扮靓。

　　13、抓好影响油田和城市安全的占压油气管线建筑动迁。大庆现有375万平方米房屋建筑占压油气管线，是油田和城市安全的重大隐患。把解决占压油气管线问题摆上重要议事日程，深入研究解决方案。积极向国家申请，争取专项资金支持。按照先易后难、先急后缓的原则，分期分批动迁。今年争取动迁改造30万平方米以上，力争用5年时间基本解决占压油气管线问题。

　　社会事业要以“大文化”建设为统领，以分离大企业办社会为契机，以市场化、产业化为方向，全面加快社会事业发展。

　　14、抓好分离大企业办社会职能的移交和整合。争取把大庆纳入国家分离企业办社会职能试点城市，获得专项政策支持。本着成熟一块剥离一块的原则，积极稳妥推进。对于暂不能分离的，帮助大企业进一步优化资源配置，逐步实现社会化管理和市场化经营。

　　15、抓好引进大学、实验中学迁建和提升基础教育水平的工作。制定引进大学的规划和支持高等教育发展的政策办法，引导大庆高等教育快速健康发展。支持石油学院向综合性大学发展，大庆高专专升本后建设高水平师范院校，大庆职业学院创办国家级示范性学院。继续建设八一农大二期、哈医大大庆校区、东北林大大庆生物技术研究院、哈工大大庆工业技术研究院和实验中学新建工程等争取今年10月前交付使用。抓好教师队伍建设，提高幼儿教育、义务教育和普通高中教育质量。采取有效措施，切实提高农村教育水平。实验中学、铁人中学等争取首批晋升国家级示范高中；十七中、肇州二中等争取列入省级示范高中。大力推进职业教育，支持一些县区变普通高中为职业高中，培养学生的就业能力和经济技术实用人才。

　　16、抓好疾病控制体系和公共卫生体系建设。重点建好疾病预防控制中心和疾控预警信息网络，提高重大疾病综合防治、突发性卫生事件快速反应和疾病信息检验监测能力，及时防大病、抗大疫。支持各医疗机构与国内外名院名科合作，建设一批国际先进、国内一流的医疗中心。开展结对支援、村医拜师等活动，重视乡村防疫保健工作，提高基层医疗水平。

　　17、抓好活跃市民文化生活的特色文化精品建设。有效整合文化体育资源，组织开展具有大庆特色的系列文体活动，凝聚人气，鼓舞斗志，激励创业。与大企业共同举办首届大型文艺汇演，通过多种方式展示大庆精品文化。办好“大庆之冬”艺术节，开展冰雪游乐、广场文化、百团百场演出等群众文化活动，营造特色浓厚的大庆文化氛围。有效利用体育场馆设施，举办专业比赛，开展群众体育活动。抓好铁人纪念馆新馆、文化艺术中心项目的开工，积极促进科技馆、博物馆新馆、图书城、奥体中心、篮球俱乐部训练基地等文化体育项目的招商引资工作，加快大庆文化体育事业发展。

　　关心群众生活要从解决就业、低保、就医、扶贫、减负、用水等具体问题入手，干群众赞成的事，办市民受益的事。

　　18、抓好就业和再就业工程。通过开发项目、主辅分离、发展第三产业和民营经济、置换购买、劳务输出等途径，开发就业岗位3．9万个，努力扩大就业。采取定向培训、按需培训、定单培训等方式，加强对下岗失业人员的就业培训和农民的技能培训。在饮食服务业推行就业资格证制度，确保培训质量。落实再就业政策，降低再就业成本。畅通劳动力供求信息渠道，提高就业服务水平。增强就业和再就业的组织化程度，加快农村劳动力转移。打击非法用工，整顿劳动力市场秩序，重点解决工资拖欠问题，维护劳动者合法权益。

　　19、抓好特困群体救助。本着帮贫向就业转移、救济向保障转轨、实物向货币转化的原则，完善特困群体“七位一体”救助办法。适当提高四县低保标准，全面落实社保政策。建立困难职工帮扶中心，介绍就业岗位，组织就业培训，提供小额创业贷款。扎实有效地开展城市“一帮一”和农村“包帮扶”活动。落实农民减负政策，切实减轻农民负担。启动城市低保人群和国有关停、破产企业退休人员医疗保险，研究制定低保人群大病救助办法。区级医院逐步建成面向特困群体和低收入人群的爱心医院。

　　20、抓好城市供水和农村人畜饮用水工程。研究北引扩建、南引扩容、中引利用方案设计，启动建设冯家围子水库，提高城市供水总量。用3年时间完成主城区重点区域供水管网改造，有效解决工业用水和生活用水。实施国家农村饮水解困工程计划，抓好农村人畜饮用水工程。启动肇州、肇源老区饮水解困项目，建设农村饮水工程252处，解决13．7万人饮水困难。

　　三、强化求真务实作风，进一步提高政府工作的效能和水平

　　第一，扩大对外开放，拓宽发展空间。今年是全市“招商引资年”和“改善环境年”。要围绕大力发展非油产业和非国有经济，加快引进项目、资金、技术和人才，扩大对外经贸合作，全面提升对外开放层次和水平。引进大项目。依托大企业的大资产、大原料、大市场，推出一批发展接续产业的立市大项目，吸引外商直接投资或股权并购，引进战略投资者，力争在与国内外500强企业联合上实现零的突破。盯住俄、日、韩等国家和上海、大连、温州等城市，进行重点招商。争取引进40个亿元以上项目，到位资金确保50亿元，力争100亿元。培育大集团。组建大庆对外经济贸易（集团）股份有限公司，争取原油、成品油进出口经营权。抓好晟凯毛毯、金锣肉制品、三胜塑料编织袋等出口项目。支持石油石化大企业开拓国际市场，发展跨国经营业务。实际利用外资2060万美元以上，外贸进出口总额确保8560万美元，力争突破1亿美元。构筑大通道。变驻外办事处为经贸办事处，变接待为招商。在绥芬河、黑河等口岸城市设立外联办事处，在俄罗斯、中东、美国、日本、韩国设立大庆经贸联络处，发展与海外中资公司、中介机构、华侨社团、经济组织的联系与合作，拓宽大庆对外经贸通道。

　　第二，推进改革创新，激活发展动力。坚持市场化改革取向，注重制度建设和体制创新，为经济发展注入强大动力。深化石油石化大企业改革。支持帮助大企业建立现代产权制度。支持帮助存续企业在划小核算单位、实施独立经营的基础上，逐步实现股份制和混合所有制改造，为引进战略投资者实施嫁接重组创造有利条件。研究制定工商登记、土地处置、税收等扶持政策，降低大企业改革成本，焕发大企业在老工业基地振兴中的巨大潜能。深化财税体制改革。扩大财政集中收付范围，对未纳入集中支付的单位依法逐步纳入。完善政府采购制度，将工程和服务纳入政府采购。强化税收征管，实行纳税信用等级评定制度。扩大统一有奖发票范围，向洗浴、美容美发、健身娱乐等服务领域延伸，增加零散税源收入。深化社会保障制度改革。抓住国家把黑龙江省列为社会保障制度改革试点单位契机，进一步完善社保体系。逐步将城市从业人员整体纳入社会保险，扩大社保覆盖面。深化事业单位改革。今年是事业单位改革突破年。按照“脱钩、分类、放权、搞活”的方针，经费实行财政包干，用人实行合同制、竞聘制，把用人和分配的权力下放到基层，把企业化管理的事业单位推向市场。

　　第三，密切地企协作，增强发展合力。真心实意支持大企业、服务大企业、联合大企业。经济发展齐抓。支持油田企业参与境外油气开发，拓展发展空间。支持石化企业搞好项目开发，壮大化工产业规模，提高产业层次。运用市场经济办法，加速存续企业与地方经济互补融合。政策项目合争。主动与大企业联手协作，重点向中油集团争取新的立市大项目、建立接续产业发展专项资金、分离企业办社会职能、占压油气管线建筑拆迁等方面的政策和项目，使政策更多地向大庆倾斜，项目更多地向大庆摆放，资金更多地向大庆投入。城市环境联建。按照东城区东移北扩、西城区中联西拓的布局，共同做好世纪大道沿线城市设计等26项城市建设规划。积极协调大企业在城市绿化、泡泽治理、污染防治、基础设施建设等方面继续加大投入，共同建设美好家园。社会稳定共促。搞好油田周边治安秩序整治，清理生产区内违法违章建筑，严厉打击盗抢原油和油田生产物资等违法犯罪活动，严密防控各种不稳定因素，营造良好的生产和生活环境。

　　第四，壮大民营经济，培植发展力量。毫不动摇地鼓励、支持和引导民营经济发展，释放民资民智民力。建设载体。提升完善太阳升原粮批发市场、绿色食品展销中心等一批专业市场，培育发展特色园（街），搞好配套设施建设。抓好各类创业广场、民营科技示范区建设，集中发展，规模推进，为民营经济提供更大的发展空间。放活政策。充分发挥中小企业局民营企业“代言人”作用，支持民营企业通过租赁、承包、参股、收购等多种方式，参与国企重组改制。鼓励民间资本进入基础设施、新型服务业、公用事业、外经贸、股份制银行等领域，在项目提供、资金融通、信息服务、人才培训、土地使用等方面，民营经济与其它所有制经济一视同仁。建立民营企业信用体系，为民营企业发展创造良好的融资环境。产业升级。支持民营企业向科技型、外向型发展，提高竞争力。结合老工业基地改造，引导民营企业开发一批项目，对接一批项目，形成新的竞争优势。重点抓好金锣、太福、日月、大丰、晟凯等民营企业大户，促其加大投入，加快项目开发，规模发展，培育一批产值十几亿、几十亿的民营企业集团。

　　第五，加强精神文明和法制建设，提供发展保障。以创建全国文明城市为牵动，开展系列群众性精神文明创建活动，弘扬大庆精神、铁人精神，不断提高城乡文明程度和市民文明素质，力争首批进入全国文明城市行列。围绕影响改革发展稳定的症结问题，深入开展“百事剖析、百题突破”活动，推动思想大解放，塑造与时俱进的大庆人。全力实施人才强市战略，采取更加开放的态度，更加优惠的政策，更加到位的服务，着力培养、引进、建设一批高素质的行政管理队伍、企业家队伍、科技队伍和实用人才队伍。办好博士后工作站、留学生创业园，建设科学家村，营造有利于人才成长和干事创业的激励机制、社会条件和人文环境。推进“十二”进社区，完善社区功能，加快社区建设向新建小区、老居住区、县城和中心村镇延伸，加强老年活动场所等服务设施建设，创建服务型、学习型、平安型、康乐型社区。重视民族宗教工作，抓好军警民共建，做好优抚安置。深入开展“四五”普法，推进依法治市，增强全民法制观念和法律素质。严厉打击各种刑事犯罪，扫除“黄赌毒”等社会丑恶现象，进一步防范和处理好“法轮功”等邪教问题，有效预防和及时处置群体性事件，坚决防止重特大安全事故发生，打造“平安大庆”。

　　第六，坚持执政为民，优化发展环境。以实施《行政许可法》为契机，创新行政管理，推进依法行政，建设亲民、务实、清廉、法治政府，大力改善经济发展环境。转变职能，强化服务。完善市长信访接待日和市长热线制度，政府部门全部开通行政首长公开电话。实行新闻发布、决策听证制度，保障全市人民对政府工作的知情权、参与权和监督权。完善政府超市服务工程，公布新一轮行政审批清理事项，进一步规范行政审批。全面落实“三先三后、四个零”优惠政策，营造亲商、靠商、护商、富商的发展环境。发挥政府宏观调控职能，加强市场监管，整顿市场秩序，净化法治环境。依法行政，廉洁自律。自觉接受人大法律监督和政协民主监督，主动听取各民主党派、工商联和社会各界人士意见，支持工会、共青团、妇联等人民团体工作。规范政府施政行为，提高行政执法水平。坚持重大决策集体讨论，严格按程序办事，推进决策民主化、科学化。认真落实党风廉政建设责任制，从制度建设入手，强化教育、惩治和监督，查办大要案，纠正部门和行业不正之风。落实责任，改进作风。树立科学的发展观，确立以项目为中心的工作导向，实行重点项目市、县（区）领导分包目标管理责任制。完善联席协商、现场调度、重点项目“直通车”和新闻媒体排行榜制度，推进项目落实。要深入体察民情，充分了解民意，广泛集中民智，想实招、办实事、求实效。教育广大公务员正确对待权力、地位和利益，始终保持严谨的作风、严明的纪律、严格的要求，始终保持昂扬向上的精神状态、奋发有为的开拓意识、只争朝夕的工作干劲。不讲条件，多干工作；不争待遇，多做贡献；不论个人得失，多想群众利益，为党和人民的事业鞠躬尽瘁，竭尽全力。

　　各位代表，大庆已经进入全面振兴发展的新时期，让我们在市委的领导下，高举邓小平理论伟大旗帜，坚持“三个代表”重要思想，大力弘扬求真务实精神，大兴求真务实之风，同舟共济，负重奋进，全面做好今年的各项工作，为建设更加文明、更加富裕、更加开放、更加美丽的大庆努力奋斗！
